# Assessment of Nutritional Status of Under-Five Children in an Urban Area of South Delhi, India

**DOI:** 10.7759/cureus.34924

**Published:** 2023-02-13

**Authors:** Mohit Goyal, Nidhi Singh, Richa Kapoor, Anita Verma, Pratima Gedam

**Affiliations:** 1 Community Medicine, Vardhman Mahavir Medical College and Safdarjung Hospital, New Delhi, IND

**Keywords:** nutritional status, delhi, under 5 children, ciaf, wasting, stunting, underweight, malnutrition

## Abstract

Introduction

Malnutrition among children continues to be a severe public health problem worldwide, whether in a developing country like India or a developed nation. Correct estimation of the problem is a prerequisite to planning the measures to control it.

Objective

To estimate the prevalence of undernutrition among children under five years of age by utilizing the Composite Index of Anthropometric Failure and the WHO growth charts.

Methods

From January to March 2020, 1332 children under the age of five years participated in a facility-based, descriptive, cross-sectional study at Fatehpur Beri, Urban Primary Health Center. An anthropometric assessment for each participant was done as per the WHO criteria. The data were entered into a Microsoft Office Excel spreadsheet (Microsoft Corporation, Redmond, WA) and analyzed with WHO Anthro software (WHO, Geneva, Switzerland) and a licensed version of SPSS 21 (IBM Corp., Armonk, NY). Continuous data were expressed using appropriate measures of central tendency, while categorical data were expressed in either frequency or proportions.

Results

The mean age of the study participants was 23.04 ± 18.24 months, and males (53.3%) were more than (46.7%) females. The prevalence of being underweight was 24.5% (327/1332), of which 24.1% (79/327) of children were severely underweight. Of the total study participants, 27.3% (362/1332) were stunted, and 17.8% (237/1332) were wasted, of which 29.1% (69/237) were severely wasted. The prevalence of anthropometric failure was 45%.

Conclusions

According to the findings of this study, the prevalence of undernutrition among the study participants was substantial. Furthermore, considering weight for age as the sole criterion may underestimate the true prevalence of malnutrition. The findings have critical implications for future interventions and initiatives among children in India.

## Introduction

Malnutrition among children under five years is a significant public health problem. According to the World Health Organization (WHO), malnutrition means deficiencies, excesses, or imbalances in a person's energy or nutritional consumption [1]. The term malnutrition refers to two distinct groups of conditions. The first is undernutrition, which includes being underweight (low weight for age), stunting (being short for age), wasting (being underweight for height), and nutritional deficiencies or inadequacies such as lack of essential vitamins and minerals. The second aspect refers to individuals being either overweight or obese [1,2].

Worldwide, approximately 1.9 billion adults are overweight, while 462 million are underweight. Overweight or obese children under the age of five years are estimated to number 41 million, with 159 million stunted and 50 million wasted [1]. Undernourished children have a higher risk of death and are more likely to contract childhood illness [3-5]. They are prone to be cognitively impaired, perform worse in school, have lower earning potential, and are at a higher risk of developing non-communicable diseases later in life [6]. The consequences of poor nutrition begin in utero and last for generations [7]. Undernourished women are more likely to have low birth weight babies, who are more likely to have suboptimal growth and development [8]. In response to this evidence, the WHO has set goals to reduce the number of stunted children by 40% and maintain childhood wasting to less than 5% by 2025 [9,10]. The United Nations (UN) adopted the first-ever UN Decade of Action on Nutrition to accelerate this process from 2016 to 2025 [10]. In support of this, goal 2 of the Sustainable Development Goals (SDGs) also purports to end hunger by 2030 [10]. Several nutrition targets were agreed upon in the years running up to 2016. To date, however, most of these targets remain unmet.

As per the WHO estimates, by 2025, the number of stunted children worldwide will reach 131 million (27 million above the expected 40% reduction in the target number of stunted children), while the prevalence of wasting will remain well above the 5% target [9]. The majority of the global burden of childhood undernutrition remains concentrated in low-income and lower-middle-income countries [11,12]. In India, according to the National Family Health Survey 5 (NFHS-5), in 2019-2020, 32.1% of children under five years of age were found to be underweight, 19.3% were wasted, and 35.5% were stunted [13]. As per the NFHS-5 data, the prevalence of underweight and stunting in Delhi was 21.2% and 30%, respectively [13]. The current under-five mortality rate in India is 28/1000, and undernutrition is one of the significant contributors to under-five mortality in India [14].

Weight-for-age (WFA), height-for-age (HFA), and weight-for-height (WFH) are the primary indicators used for measuring undernutrition [15]. However, in a population, different degrees of overlap between these indicators are noticed, i.e., some underweight children might also be stunted or wasted, and some children might have all three forms of anthropometric failure, i.e., stunting, wasting, and underweight. To measure the prevalence of undernutrition more precisely and accurately in a population, Svedberg developed the Composite Index of Anthropometric Failure (CIAF) [16]. In India, Nandy et al. were the first to use the concept of the CIAF on the 1998-1999 National Family Health Survey-2 (NFHS-2) data [17]. Hence, to compare the utility of the CIAF and the WHO Z-score classification, the present study was planned to determine the overall prevalence of undernutrition status among children under five years residing in an urban area of Delhi.

## Materials and methods

Study setting and duration

This was a cross-sectional descriptive study conducted at the Fatehpur Beri Urban Primary Health Center (UPHC) in South Delhi, which caters to a population of approximately 58,000 and falls under the South Delhi Municipal Corporation (SDMC). Children under five years contribute approximately 25% of the monthly outpatient department (OPD) cases at the center. The study duration was of three months (from January 2020 to March 2020).

Sampling methods

A complete enumeration of all children under five years visiting the UPHC between January 2020 and March 2020, seeking immunization and healthcare services, was done. Children under the age of five years (0-59 months) are periodically monitored for growth parameters at the UPHC in accordance with programmatic guidelines under the Reproductive, Maternal, Newborn, Child, and Adolescent Health (RMNCH+A).

Sample size

Of the 1450 potential participants identified, 1332 agreed to participate, yielding a response rate of 91.9% (1332/1450) during the three-month study period.

The inclusion criteria included healthy children under five years of age and participants who were willing to participate, i.e., whose parents gave written informed consent to be a part of the present study.

The exclusion criteria included children born with congenital diseases or currently undergoing treatment for chronic illnesses.

Data collection

Parents of the selected participants were interviewed by a trained team of doctors using a semi-structured questionnaire seeking information on the socio-demographic characteristics of the participants.

Anthropometric measurements such as weight and height were recorded following the WHO guidelines [15]. Indicators based on weight, height, and age were further assessed and compared with the WHO growth reference standards (2006) and CIAF to assess the nutritional status of children [16].

Weight

The participant's weight was measured in kilograms with a weighing scale to assess their growth and nutritional status using the standard technique to the nearest 0.5 kg. If the child was less than two years old or was unable to stand, tared weighing was performed, and if the child was two years or older, the child was weighed alone using a standardized, recently calibrated analog weighing machine.

Height

Using the standard technique, the participant's height was measured using a stadiometer/infantometer to the nearest 0.1 cm. If a child was less than two years of age, recumbent length (lying down) was measured using an infantometer; however, if the child was two years or older and could stand, standing height was measured using a stadiometer.

Operational definitions

Underweight

The participant's weight was recorded and compared to the median values; alternatively, the participant's weight was plotted against age on a graph for comparison with the standard curve. A low weight-for-age is termed as underweight, defined as a weight-for-age Z-score (WAZ) of less than -2. Severely underweight is classified if WAZ is less than -3 of the WHO (2006) reference values [15].

Wasting

Wasting is an indicator of acute malnutrition and is defined as a weight-for-height Z-score (WHZ) of less than -2. A Z-score between -2 and -3 is classified as moderate wasting. Severe wasting is classified if WHZ is less than -3 according to the WHO (2006) reference standards. The data collected were entered into the WHO Anthro software (WHO, Geneva, Switzerland) for analysis. The prevalence of undernutrition was determined using the CIAF and the WHO Z-scoring systems.

Stunting

Low height for age indicates stunting and depicts early chronic exposure to undernutrition. Stunting is a height-for-age Z-score (HAZ) of less than -2. A Z-score between -2 and -3 is considered moderate stunting, and severe stunting is classified if HAZ is less than -3 of the WHO (2006) reference standards.

CIAF

The under-nutritional status of children was also classified based on the CIAF using Nandy et al.'s model of six groups (Table 1) [17].

**Table 1:** Classification and interpretation of the CIAF categories CIAF: Composite Index of Anthropometric Failure.

Category	Interpretation
A	No failure
B	Wasting only
C	Wasting and underweight
D	Wasting, stunting, and underweight
E	Stunting and underweight
F	Stunting only
Y	Underweight only

## Results

Socio-demographic characteristics of the study participants

**Figure 1:**
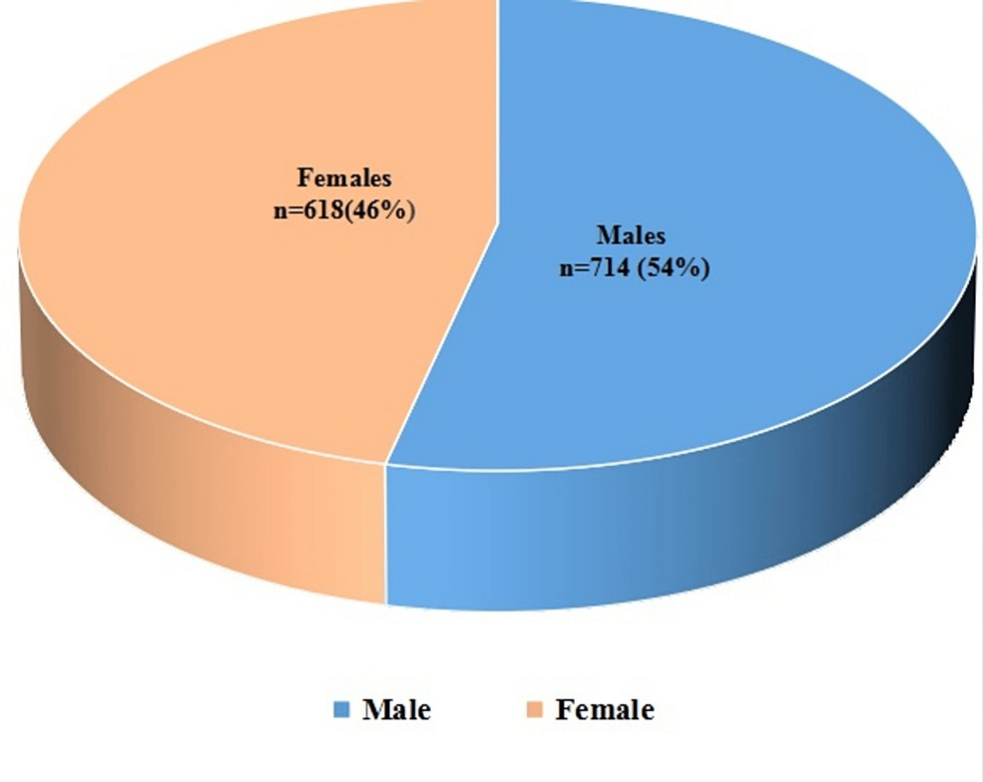
Distribution of the study participants by gender (N = 1332)

A total of 1332 children were assessed to collect baseline data. More than half of the participants were males (54%, n = 714), and the rest were females (46%, n = 618) (Figure 1). There were 457 (34.3%) children aged less than one year, 293 (22%) children aged 12 months to 23 months, 187 (14%) children aged between 24 and 35 months, 163 (12.2%) children were in the category of 36-47 months, and 232 (17.4%) children aged between 48 and 59 months (Table 2).

**Table 2:** Distribution of the study participants by age and nutritional status (N = 1332) CIAF: Composite Index of Anthropometric Failure.

Age in months	Category			
	Stunted, n (%)	Underweight, n (%)	Wasted, n (%)	CIAF, n (%)
0-5 (n = 287)	38 (13.2)	63 (22.0)	81 (28.2)	130 (45.3)
6-11 (n = 170)	30 (17.6)	43 (25.3)	40 (23.5)	75 (44.1)
12-23 (n = 293)	98 (33.4)	58 (19.8)	40 (13.7)	128 (43.7)
24-35 (n = 187)	83 (44.4)	57 (30.5)	25 (13.4)	61 (54.0)
36-47 (n = 163)	48 (29.4)	45 (27.6)	18 (11.0)	68 (41.7)
48-59 (n = 232)	65 (28.0)	61 (26.3)	33 (14.2)	98 (42.2)
Total (N = 1332)	362 (27.2)	327 (24.5)	237 (17.8)	600 (45)

Nutritional assessment according to the WHO Z-scores

**Figure 2:**
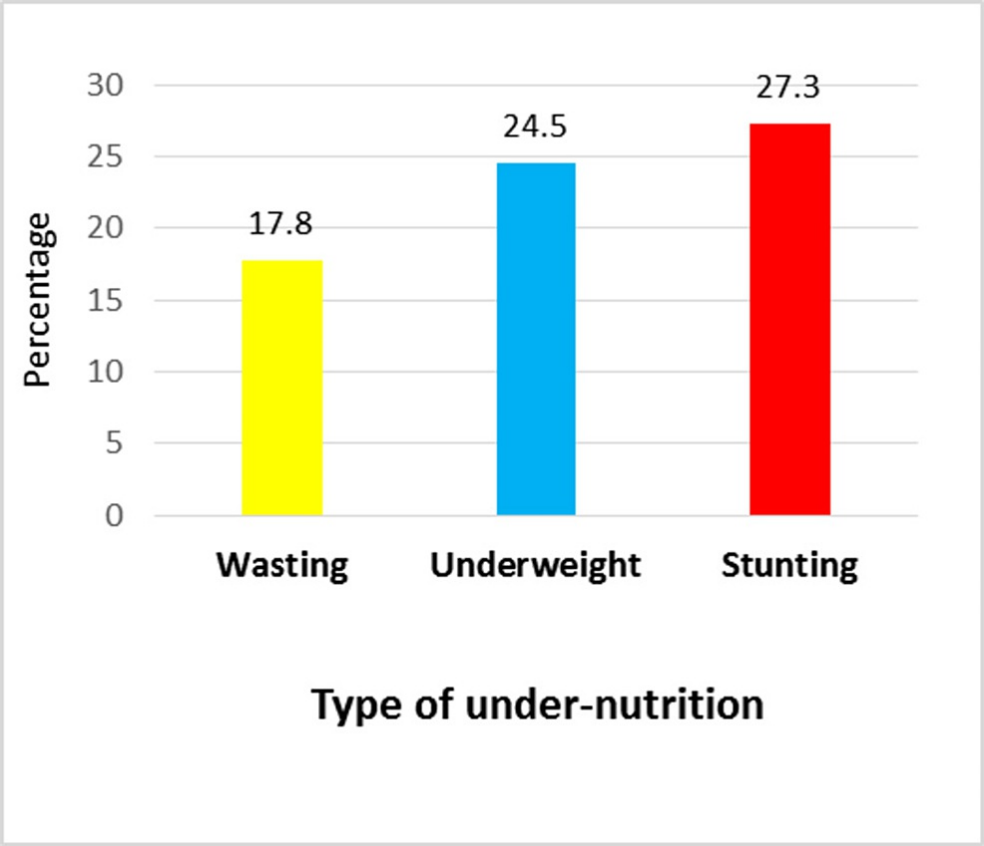
Prevalence of undernutrition among the study participants (N = 1332)

In the current study, the prevalence of underweight, stunting, and wasting was 24.5%, 27.3%, and 17.8%, respectively (Figure 2). Among those underweight, a quarter (24.1% ) of children were severely underweight (WFA z < -3SD) (Table 3).

**Table 3:** Nutritional assessment of the study participants based on the WHO categories (N = 1332)

Category	Underweight, n (%)	Wasting, n (%)	Stunting, n (%)
Severe	79 (5.9)	69 (5.2)	136 (10.2)
Mild/moderate	248 (18.6)	168 (12.6)	226 (17.1)
Normal	1005 (75.5)	1095 (82.2)	970 (72.7)
Total	1332 (100)	1332 (100)	1332 (100)

Nutritional assessment according to the CIAF

On applying the CIAF, almost half (45%) of the study participants were undernourished. As per the CIAF, 55% of children fell in category A (no failure). Exclusive categories like B (wasting only), F (stunting only), and Y (underweight only) had a prevalence of 7.65%, 12.8%, and 3.75%, respectively. Nearly one-fifth (20.8%) of the study participants had different combinations of undernourishment as demonstrated by category C (wasting and underweight), category D (wasting, underweight, and stunted), and category E (stunting and underweight) of the CIAF (Table 4).

**Table 4:** Prevalence of undernutrition based on the CIAF among children under five years (N = 1332) CIAF: Composite Index of Anthropometric Failure.

CIAF category	Anthropometric status	Number of children (%)
A	No failure	732 (55%)
B	Wasting only	101 (7.7%)
C	Wasting and underweight	87 (6.5%)
D	Wasting, underweight, and stunted	48 (3.6%)
E	Stunting and underweight	143 (10.7%)
F	Stunting only	171 (12.8%)
Y	Underweight only	50 (3.8%)
CIAF (B + C + D + E + F + Y)		600 (45%)

Factors associated with undernutrition

Out of the 498 study participants who were >24 months old, 151 (30.3%) were stunted, whereas out of a total of 834 participants between zero and 24 months, 211 (25.3%) were stunted and the difference between the two proportions was statistically significant (p < 0.05). Significantly, a higher proportion of participants between zero and 24 months when compared to those >24 months in age were found to be wasted. This difference was found to be highly significant for both groups (p < 0.05). As the age of the study participants increased, the prevalence of stunting increased, peaking at 44.9% among children aged 24-35 months. The prevalence of underweight, wasting, and stunting was higher in males than females; however, this difference in proportion across genders was not found to be statistically significant (p > 0.05) (Table 5).

**Table 5:** Association of age and gender with nutritional status among the study participants (N = 1332) * Significant at p-value < 0.05.

Variable	Underweight		P-value	Stunting		P-value	Wasting		P-value
	Yes, n (%)	No, n (%)		Yes, n (%)	No, n (%)		Yes, n (%)	No, n (%)	
Age									
0-24 months	193 (23.1)	641 (76.9)	0.12	211 (25.3)	623 (74.7)	0.04*	174 (20.9)	660 (79.1)	<0.01*
>24 months	134 (26.9)	364 (73.1)		151 (30.3)	347 (69.7)		63 (12.7)	435 (87.3)	
Gender									
Male	186 (26.1)	528 (73.9)	0.17	195 (27.3)	519 (72.7)	0.90	133 (18.6)	581 (814)	0.39
Female	141 (22.8)	477 (77.2)		167 (27)	451 (73)		104 (16.8)	514 (83.2)	

## Discussion

In the current study, the prevalence of underweight, stunting, and wasting was 24.5%, 27.2%, and 17.8%, respectively. These findings are comparable to the national prevalence of 32.1%, 35.5%, and 19.3% of underweight, stunted, and wasted, respectively, as per the NFHS-5 data [13]. As per the NFHS-5 data, the prevalence of underweight and stunting for Delhi was 21.2% and 30%, respectively; these are comparable to the prevalence rates of the current study. However, wasting was prevalent among 17.8% of the participants in the current study, which is higher than the 11.2% reported by NFHS-5 data in Delhi [13].

The current study also used the CIAF to evaluate the nutritional status of participants. The prevalence of undernutrition, as per the CIAF, was 45%. Almost half of the study participants had some form of anthropometric failure, whereas 55% experienced no anthropometric failure (Table 4). The current study's findings are comparable with the study conducted by Ramkumar et al. (2018) in Puducherry, wherein the prevalence of CIAF was 48.7% [18]. However, the prevalence in the current study is higher than the 36.1% and 32.7% reported by Roy et al. (2014) and Dasgupta et al. (2014) from rural areas in West Bengal [19,20]. This difference can be attributed to the fact that the study settings (urban vs. rural) were distinct, and a different set of risk factors may have played a role and contributed to the observed difference from the present study. However, the prevalence of 45% in the current study is much lower than the 62.5% reported by Anwar et al. (Varanasi) and 58.6% reported by Dhok et al. in Nagpur [21,22]. It was also lower than the prevalence of 63.6% found by Sen et al. in Darjeeling [23]. Damor et al. reported the prevalence of malnutrition to be 54% among children aged between one and five years [24]. However, the difference can be attributed to the different methodologies used to classify malnourished, i.e., Gomez's classification in their study, which took into account only weight for age criteria.

The individual criteria for undernutrition in the current study revealed the prevalence of underweight (WFA < -2SD), stunting (HFA < -2SD), and wasting (WFH < -2SD) to be 24.2%, 27.5%, and 17.4%, respectively (Tables 2, 3). The findings imply that the prevalence of undernutrition was substantially lower than that of CIAF when individual criteria were considered, i.e., 45%. Similar findings have been reported by prior research. Roy et al. (2014), in a rural area of West Bengal, reported CIAF to be 36.1%, and the prevalence of stunting, underweight, and wasting was 16.7%, 29.2%, and 22.2%, respectively [19]. Sen et al. (West Bengal, 2012) found the CIAF to be 63.6%; however, individual parameters were 52% (underweight), 43.3% (stunting), and 21.5% (wasting), which is lower when compared to the prevalence of CIAF [23]. Nandy et al. also revealed that the prevalence of underweight was 47.1%, while CIAF was 59.9%, which was much higher than the individual criteria [17]. Similarly, CIAF was 65.3% for toddlers in a study by Seetharaman et al., and the prevalence of underweight was 46.6%, which is much lower than the CIAF [25].

The findings also suggest no significant difference existed in childhood undernutrition across genders. However, children in the younger age group (0-24 months) had a higher risk of being wasted, whereas a higher proportion of children >24 months in age were more likely to be stunted when compared to children in the younger age group. This finding is explained by the fact that wasting is a sign of acute malnutrition, whereas stunting represents chronic malnutrition, which takes time to exhibit symptoms.

The CIAF is a comprehensive tool permitting the segregation of undernourished children into different subgroups for further analysis. From Table 4, we observe that 45% of the children suffered from one or the other form of anthropometric failure. We can identify 25% of the children from subgroups C, D, E, and Y by using low weight for age (underweight) as the only criterion for undernourishment; however, we miss the children in subgroups B and F who were stunted and wasted but not underweight in the current study. Therefore, 20% of such children would be missed out as not being undernourished. Similarly, stunting misses groups B, C, and Y (28% of children), and wasting misses those children in groups E, F, and Y (27% of children).

The present study had the following strengths: as the study was conducted under routine programmatic conditions, a large sample size was collected within a short period. Secondly, comprehensive standard operative procedures were established for undertaking anthropometric measurements reducing measurement bias. Both the CIAF and the WHO growth charts tools were used to assess nutritional status allowing for comparisons and inferences to be drawn simultaneously. All severely undernourished children were referred to the nearest nutritional rehabilitation centers, and follow-up visits were undertaken for the mild to moderately undernourished. Nutritional counseling sessions were conducted for all parents, along with line list preparations by Anganwadi workers (AWWs), Accredited Social Health Activists (ASHAs), and auxiliary nurses and midwives (ANMs).

This paper has some limitations. Firstly, because the study was cross-sectional, causal inferences cannot be drawn. Second, the survey was limited to children from a single UPHC in a particular state; therefore, incorporating children from other primary health centers (PHCs) in different locations may yield different results. As a result, these results cannot be generalized to the entire state or country. Third, aside from gender and age, other potential risk factors for malnutrition were not captured. Fourth, the children were not followed up; thus, a longitudinal study may be more beneficial. Researchers verified that all anthropometric measures were precise to prevent measuring bias. Nonetheless, the current study's findings are consistent with previously published literature.

## Conclusions

The prevalence of undernutrition was high among the study participants. The CIAF is a comprehensive tool for calculating the total number of undernourished children in a community in terms of a single composite number. The CIAF enables the early identification of children with numerous anthropometric failures by categorizing undernourished children into several different groups. Hence, it enables healthcare providers to prioritize and provide swift treatment and care to those who need it the most. This information can further enable healthcare workers, clinicians, planners, and policymakers better estimate the prevalent problem in the community allowing the implementation of need-specific reforms, interventions, and policies.

More studies utilizing the CIAF are required among children from other areas of Delhi, as well as from other parts of India, to acquire a broader representation. These findings will not only allow us to compare the rates of three standard indicators of undernutrition with CIAF but will also aid in establishing CIAF's increased effectiveness and utilization. Future studies should also focus on a comprehensive exploration of risk factors contributing to the burden of malnourishment among this age group.
